# Sociodemographic and Lifestyle Factors Associated with Adherence to Mediterranean Diet in Representative Adult Population in Casablanca City, Morocco: A Cross-Sectional Study

**DOI:** 10.1155/2020/3105271

**Published:** 2020-03-20

**Authors:** Karima Mohtadi, Rajaa Msaad, Najwa Benalioua, Ali Jafri, Hasnaa Meftah, Younes Elkardi, Halima Lebrazi, Anass Kettani, Abdelfettah Derouiche, Hassan Taki, Rachid Saïle

**Affiliations:** Laboratory of Biology and Health, URAC 34, Faculty of Sciences Ben Msik, Health and Biotechnology Research Center, Hassan II University of Casablanca, Casablanca, Morocco

## Abstract

Several studies had revealed that following the Mediterranean diet (MD) contributes to beneficial health status and a decreased risk of many chronic diseases. The aim of our study was to assess adherence to MD in Casablanca City and to identify the relationship between MD adherence and sociodemographic and lifestyle parameters. This cross-sectional study concerned 719 subjects with complete dietary data. Data collection was performed using a questionnaire including sociodemographic and lifestyle factors. The dietary intake was assessed with the use of a food-frequency questionnaire. The compliance with MD was evaluated with a simplified MD score. Our study showed that high adherence to the MD was characterized by high intakes of vegetables, fruits, pulses, fish, cereals, olive oil, and low meat and dairy consumption according to the Simplified MD score. As regard to the multivariate logistic, being a man, being married, persons with a level of education >6 years, luxurious housing, and consumption of alcohol were associated with a higher adherence to MD, while, the overweight was negatively associated with a higher adherence to MD. Maintaining the traditional MD pattern is crucial for public health; in this way, more research is needed in this area in order to precisely measure these associations.

## 1. Introduction

Mediterranean diet (MD) is vastly considered the overall dietary habits of the Mediterranean Basin [1]. There are several variants of the MD; each Mediterranean country has its own gastronomic customs influenced by various factors such as economic, sociocultural, and religious factors [2]. Indeed, this diet is characterized by some common features: high intake of fruits, nuts, vegetables, legumes, cereals (including bread), and fish, olive oil as the main source of fat, moderate intake of dairy products, eggs, and poultry, moderate intake of alcohol (mainly wine during meals), and low intake of sweets, red meat, and its derivatives [3].

Epidemiological, biochemical, and clinical research has provided a solid biologic foundation for the health advantages of the MD adherence, and it was associated with intakes of many nutrients: fiber, carbohydrates, vitamins, and minerals such as vitamin B, calcium, magnesium, and potassium that can be taken throughout the day, the week, and occasionally and which are thought of as having beneficial effects on a wide range of physiological processes [4, 5]. The MD was associated with a low rate of many chronic diseases such as cardiovascular disease [6, 7], diabetes [8], obesity [9], and metabolic syndrome [10]; also, there is a proposal that the MD could have some favorable effect on cancer risk [11, 12].

Due to manifold factors such as globalization, economic development, changes in food consumption, and the adoption of unhealthy lifestyles, the traditional MD is now progressively eroding in Mediterranean countries and switching to westernized diet [1, 4]. Foods native to the Mediterranean area have been modified over time with the incorporation of new foods and methods of preparation; some have enriched and others have worsened the traditional MD [13].

Morocco, like several developing and Mediterranean countries, is suffering from the consequences of a deviation from the MD, and it has shown much progression in the last decades [14]. Its dietary patterns incurring profound and rapid modifications as a consequent of growing urbanization, economic development, globalization, and increased production in the food industry, thus, conduct to a nutritional transition [15].

To evaluate adherence to MD, numerous indexes or scores have been created. Hence, the index or scores summarize the diet by means of a single score that results from a function of different components, such as food, food groups, or a combination of foods and nutrients [16]. The first and most broadly used is the Mediterranean Dietary Score (MDS), proposed by Trichopoulou et al., which assesses the compliance with this MD in adults, including the elderly, using as cutoffs the sex-specific medians, attributing 1 point if the intake of protective foods in the MD is above the median or when the intake of non-protective foods is below the median, and 0 point in the opposite situations [17, 18]. Application of these indexes has shown modifications in dietary habits [19] and that some factors such as sociodemographic parameters, general health status, lifestyle, and psychological factors, were associated with a greater adherence to MD as elderly age [20], female sex and moderate alcohol consumption [21], country of origin [22], and the practice of physical activity [23] or were associated with poor adherence as younger populations (children and adolescents) [19, 24], higher BMI, and obesity [25]. In this context, the aim of our study was to evaluate adherence to Mediterranean diet in Casablanca City and to identify the relationship with MD adherence, demographic, socioeconomic, and other lifestyle parameters.

## 2. Populations and Methods

### 2.1. Design

The present cross-sectional study was carried out between March and June 2017 in different prefectures of Casablanca using cluster sampling. This is a random and exhaustive sample, based on the national census of 2014 and the data provided by the Higher Planning Commission (HCP). The sampling approach chosen for the survey is based on the probabilistic probing method stratified at three degrees. In the first-degree, sampling units consisted of the 2014 census districts. A sample of 80 districts was selected; their distribution was according to the districts of the city of Casablanca, and the strata of housing was carried out respecting the principle of the allocation proportional to the size in terms of number of households. In the second-degree, units consisted of households. At the level of each district taken in the first degree, 10 households were selected. The third degree: at the level of each selected household, only one adult person (male or female) was chosen to take part in the survey.

### 2.2. Study Participants

The study concerned an adult population, men and women aged 18 years and above, from different prefectures of the city of Casablanca. Pregnant and lactating women, as well as physically and mentally disabled subjects, were considered to be ineligible. All subjects gave their consent before answering the survey. This study was conducted in accordance with the Declaration of Helsinki and had the approval of the regional ethical committee.

### 2.3. Data Collection

#### 2.3.1. Data on Sociodemographic Characteristics and Lifestyle Factors

Data collection for this study was performed using a questionnaire that was inspired by the World Health Organization (WHO) instruments for chronic disease surveillance [26] and administered by trained personnel. Its face, validity was examined in a pilot study in 50 participants and showed that the questionnaire was acceptable and understandable. It included sociodemographic characteristics (age, sex, educational level, marital status, and occupation) and household characteristics (type of housing, and household size). Age was recorded according to the tertile into three categories: ≤29; 30–45, and ≥46. Educational level was grouped into three categories: illiterates, ≤6 years of schooling (primary, informal education), and >6 years of schooling (secondary, university). Marital status was classified into two classes: married and not married (single or divorced and widowed). Occupation was recorded into two groups: with a job (active or student) and without a job (retired, unemployed, and housewife). Housing was grouped into 5 classes: traditional housing, luxurious, flat, modern, and poor housing (including shantytown, room). Concerning lifestyles factors, subjects were classified according to their tobacco consumption into three groups: current smokers, ex-smokers, and non-smokers. Also, according to alcohol consumption, two categories were used: consumer and nonconsumer.

#### 2.3.2. Data on Diet

The usual dietary intake was assessed with the use of a food-frequency questionnaire including 80 foods and beverages commonly consumed in Morocco. For each of the items, respondents were asked to report their frequency of consumption over a month or over a week or a habitual day. Eventually, 14 all-inclusive foods, food groups, and beverages were considered: vegetables, legumes, fruits, dairy products (milk, yogurt, and cheese), cereals (bread, cereals, rice, pasta, and couscous), potatoes, red meat (veal, lamb, camel, and goat), white meat (poultry and turkeys), processed meat, fish, eggs, beverages (coffee, tea, and herbal infusions), sweets (Sugar, jelly, candies, pastries, soda, and sweetened fruit juices), and added fat (olive oil, other vegetable oils, and butter). The frequency of the intake of each food item was reported in daily consumption per week.

### 2.4. Anthropometric Measurements

Anthropometric measurements were collected by trained investigators in accordance with WHO standards. Weight was measured using bathroom scales. To ensure accuracy in measurement, the scale was checked for a zero reading before each weighing and calibrated with a known weight on the morning of each data collection.

The height of the participant was measured in the standing position, using a stadiometer graduated in centimeter. The participant was asked to stand without shoes and socks, with heels together and the head in the upright position.

The body mass index (BMI) (weight in kilograms divided by height in meters squared) was computed to determine overweight and obesity among adolescents using the cutoff values as recommended by the World Health Organization as follows: underweight: (BMI <18.5 kg/m^2^), normal weight (BMI ≥18.5 and <25.0 kg/m^2^), overweight (BMI ≥25.0 and <30.0 kg/m^2^), and obesity (BMI ≥30.0 kg/m^2^).

### 2.5. Simplified Mediterranean Dietary Score

To evaluate the degree of adherence to the MD, we used a simplified Mediterranean-diet score [27], and it was constructed following an adaptation of the Mediterranean Dietary Score, proposed by Trichopoulou et al. [17, 18]. The computation of the score was based on the frequency of the weekly intake of each food group; this score consists of eight components (vegetables, legumes, fruits, cereal, fish, meat, and dairy products). To calculate the total frequency of each component, we added the frequency of items that belong to it, dairy products (milk, yogurt, and cheese), cereals (bread, cereals, potatoes, rice, pasta, and couscous), and meat (red meat, white meat, and processed meat). As we could not compute the monounsaturated fatty acid to the saturated fatty acid ratio, for a fat intake, we considered olive oil intake as the main dietary source of monounsaturated fatty acid [27]. A value of 0 or 1 was contributed to each of components with the use of the sex-specific median as the cutoff. For beneficial components (vegetables, legumes, fruits, cereal, and fish), persons whose consumption was below the median were assigned a value of 0, and persons whose consumption was at or above the median were assigned a value of 1. For components presumed to be detrimental (meat and dairy products), persons whose consumption was below the median were assigned a value of 1, and persons whose consumption was at or above the median were assigned a value of 0. Hence, persons who used olive oil for dressing or for cooking were assigned a value 1 and 0 for nonconsumers.

Thus, the total simplified MD score ranged from 0 (minimal adherence) to 8 (maximal adherence). This index is then used to classify subjects into two groups according to their adherence to the MD, “low” adherence to the MD (0 to 4 points), and “high” adherence to the MD (5 to 8 points).

### 2.6. Statistical Analysis

All analyses were performed with the use of SPSS Statistical software (version 23). A descriptive analysis was conducted to compute medians and means with standard deviation (SD) for quantitative variables and frequencies (%) for qualitative variables. Categorical variables were tested using the *X*^2^ test; differences between groups were compared using Student's *t*-test.

A multivariable logistic regression was performed to analyse factors associated with following a high adherence to MD. The odds ratios (OR) were estimated, with 95% confidence intervals (95% CI) and a significance level of 5% (*P* ≤ 0.05).

## 3. Results

In total, out of the 800 people selected, 731 (91.37%) involved in the survey, with 48.2% men and 51.8% women (people who are not included in the survey had refused to participate or were absent). Of these, 12 (1.64%) individuals were expelled from the computation of the MD score; these participants had at least one missing data in at least one component of MD adherence score. Therefore, our sample consists of 719 people with complete dietary data.

The general characteristics of the study population sample are shown in Table 1. Among the 719 subjects, 373 (51.9%) were men and 346 (48.1%) were women. The mean age was 38.99 ± 15.31 years. There was a significant difference between age groups (*p*=0.005), marital status, education level, and occupation according to the gender (*p* < 0.0001). Regarding tobacco consumption and alcohol consumption, women were more likely to be nonsmokers and alcohol abstainers (93.1% and 98.0%, respectively). The overall prevalence of overweight and obesity was, respectively, 29.6% and 21.0%; also, obesity was much higher among women than men (35.0% vs. 8.0%, *P* < 0.0001).

The mean values among men and women for the dietary intake of foods and major food groups are shown in Table 2. There were significant differences among men and women in the consumption of vegetables (*P*=0.024), legumes (*P*=0.035), fish (*P*=0.001), red meat (*P* ≤ 0.001), processed meat (*P*=0.023), egg (*P*=0.022), beverages (*P*=0.026), and soda (*P*=0.016). However, no differences were found with regards to other foods. As can be seen, men consumed more legumes (mean = 4.93 weekly frequency), fish (mean = 3.73 weekly frequency), red meat (mean = 2.90 weekly frequency), processed meat (mean = 1.24 weekly frequency), eggs (mean = 4.41 weekly frequency), beverages (mean = 3.47 daily frequency), and soda (mean = 2.25 weekly frequency) than women who consumed more vegetables (mean = 5.16 daily frequency).

The mean values of simplified Mediterranean dietary score among population studied according to sociodemographic and lifestyle factors are presented in Table 3. The mean score value for the total sample was 4.79 ± 1.39. In accordance with sex, the mean value of score was 4.74 ± 1.48 in women and 4.84 ± 1.31 in men. There were no significant differences according to sex, occupation, education level, housing, smoking, alcohol consumption, and BMI class. However, a significant difference was found with regards to the age group (*P*=0.035) and marital status (*P*=0.002).

**Figure 1:**
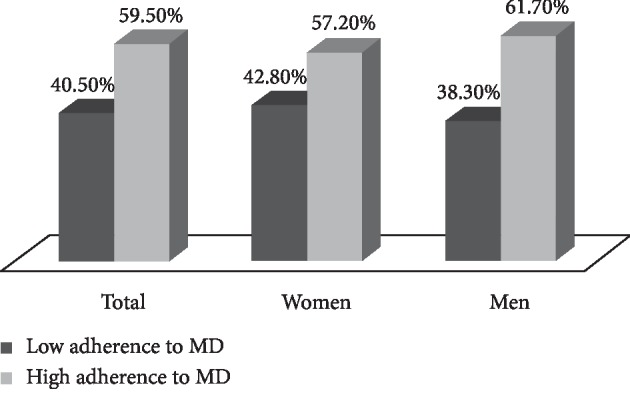
The distribution of adherence to Mediterranean diet.

The distribution of adherence according to the simplified Mediterranean dietary score (low or high adherence), subjects according to the sex and studied food groups, and using as cutoffs, the sex-specific medians are described in Table 4. Adherence to the MD was high and low for 59.5% and 40.5% in our sample (Figure 1), respectively. According to the sex, the percentage of men who had a high adherence to the MD was 61.7% (*n* = 230), while only 38.3% (*n* = 143) had a low adherence. The prevalence of women seemed to have a high and low adherence to the MD was, respectively, 57.2% (*n* = 198) and 42.8% (*n* = 148). On target, the prevalence of the intake of vegetables, legumes, fruits, cereals, fish, and olive oil increased significantly with higher adherence to the MD and decreased with lower adherence to the MD (*P* < 0.0001) in the both genders. Inversely, the consumption of meat decreased significantly (*P* < 0.0001) with higher adherence to the MD and increased with lower adherence to the MD, while for dairy products, there was no significant difference for women in the both categories of adherence to MD (*P*=0.475). Conversely to men, significant difference was found inside each group (*P*=0.009).

Median of the daily intake of food group. Dairy products (milk, yogurt, and cheese). Cereals (bread, cereals, potatoes, rice, pasta, and couscous). Meat (red meat, white meat, and processed meat). Statistically significant differences are defined as *P* < 0.05.

Food groups' consumption according to the categories of adherence to Mediterranean Diet is shown in the Table 5. According to our results, high adherence to the MD was characterized in our population by high intakes of vegetables, fruits, pulses, fish, cereals, olive oil, and low meat and dairy consumption, whereas, a low adherence to MD was characterized by low intakes of vegetables, fruits, pulses, fish, cereals, olive oil, and high meat and dairy consumption. As can be seen, there were significant differences among consumption of food group according the categories of adherence to Mediterranean diet (*P* < 0.05).

According to the multivariate logistic regression model (Table 6), the factors associated with a high adherence to MD were being a man OR = 1.46; 95% CI 1.00–2.10, being married OR = 1.46; 95% CI 1.02–2.10, luxurious housing OR = 3.74; 95% CI 1.18–11.87, high level of education (>6 years “secondary, university”) OR = 1.94; 95% CI 1.27–2.95), and consumer of alcohol OR = 2.30 95% CI 1.11–4.79, while, we found a negative association between a high adherence to MD and overweight OR = 0.59; 95% CI 0.37–1.9. Last, we must underline that there were no statistically significant associations between a high adherence to MD and the factors of age, smoking, and occupation.

## 4. Discussion

This study was conducted in Casablanca City, the economic capital of Morocco, which is the largest and the most populous city (10% of the total population) in the kingdom. The city has been facing an important increase in population (currently exceeds 3.4 million) and great urbanization (16% of the urban population of the kingdom) in the last years [28], with a population from all regions of Morocco, which allows to say that it is a representative city of the different Moroccan gastronomic cultures, and it is a city where we see a strong nutritional transition. Our study allows the assessment of adherence to MD in an adult sample of the city of Casablanca and reveals the relationship between greater adherence to MD and demographic, socioeconomic, and lifestyles factors.

Primarily, our study showed that high adherence to the MD was characterized by high intake of vegetables, fruits, pulses, fish, cereals, olive oil, and low meat and dairy consumption according to the Simplified MD score.

Indeed, according to our results, men were more likely to follow MD than women. This was in accordance with the findings of González et al. [29] who reported that MD adherence was lower in females. In contrary, Patino-Alonso et al. and Sánchez-Villegas et al. [21, 30] showed that women were more compliant than men with the MD. This could be explained by the changing role of women in the family in the last few decades. Traditional housewives have become increasingly rare, and eating outside the home has become more common.

In fact, we did not detect any association between age groups and adherence to MD, which joins a study previously carried out on our country [27], whereas, it has been reported that age was predictive factor of MD adherence in most Mediterranean regions as in Spain [19, 20], Italy [31], and Greece [24, 32]. Older subjects were more adherent to the MD than younger, may be that elderly remain loyal to the traditional lifestyle and eating habits which they grew up with and then avoid the modern dishes and fast foods, while the younger (adolescent and children) are closer to Western dietary patterns, with a reduction in the intake of some of the key foods MD, such as fruits, vegetables, and legumes, with higher consumption of fats and proteins [33, 34]. Our results are not consistent with this; this fact may be explained by cultural differences and traditional lifestyle between Moroccan people and others populations in the Mediterranean Basin; also, our sample does not include children and adolescents under the age of 18.

On the other hand, we found that the fact of being married was positively associated with the higher adhesion of the MD; the same results have been revealed in other Mediterranean countries [35, 36]. Conversely, other studies have shown [27] that single, divorced, and widowed individuals are more likely to adopt a Western diet or a poor MD. This can be related to traditional behaviour, the potential family influences, and social obligations that encourage married people to eat often in family and to prepare and share meals with family members.

Similarly, we found that highly educated subjects tended to have a higher adherence to the MD compared to those with lower levels of education, which confirms the findings of previous research that has linked higher levels of education to healthier diets and adherence to dietary recommendations [8, 9]. It can also be said that an educated population is often aware of the interest of food in the prevention of chronic diseases and that is why it adopts a healthy diet tends towards MD.

Regarding the housing classes, we have shown associations between healthier diets and people living in luxurious housing, inversely to El by Rhazi et al. [27] that showed that people living in the old and new Medina always keep their traditional way of life and the subjects living in luxurious housing tended to be associated with the lower MD adherence; our results can be explained as people of this class fall into the category of a high income level, numerous studies have shown a significant association between a healthier diet and high income levels, and consumers at this level of income can easily meet their nutritional needs and concerns about food quality and safety [37–39].

Findings relating to the alcohol consumption suggest that consumption of these beverages promotes MD adherence, and it was similar to those obtained in other studies. Bach-Faig and collaborators [4], also Patino-Alonso and collaborators [21], reported an association between consumption moderate alcohol and MD.

Regarding to the BMI classes, we have shown that the higher adherence to MD was negatively associated with overweight. Our results join the findings of previous research [40, 41].

Our study has put the point on the Mediterranean diet which constitutes a culinary heritage of Morocco and other Mediterranean countries; that tends to disappear towards a modern regime, the study of food transition in representative population of Casablanca that Morocco has known for years and allows to identify several factors related to adhesion a MD. Our study has a few limitations. The main was the frequency food questionnaire, it was semiquantitative and not quantitative; this method was originally designed to provide information about food-consumption patterns, the frequency of consumption, and servings. It is not reliable for measuring total diet, total energy intakes, and total nutrient intake, the thing which led us to use a simplified MD score [27] and not the MD score used in previous studies [17, 18]. Likewise, transversal aspect of the study does not allow us to identify the food transition especially in the new immigrants from rural areas to the urban environments of Casablanca. Thus, another limitation according to age was exclusion of children that limited the role of the age factor. The end our study remains important because it is the first that touches the dietary patterns in adult population, men and women, of Casablanca City, while other studies realized were particularly interested in women; also, our survey touched the different neighborhoods in order to broaden the study of the economic impact.

## 5. Conclusion

This study revealed that high adherence to the MD was characterized by high intakes of vegetables, fruits, fish, cereals, olive oil, and low meat and dairy consumption according to the simplified MD score. This higher adherence to MD in adult population in Casablanca City was associated with several factors such as gender, marital status, a high education level, luxurious housing, alcohol consumption, and overweight. Maintaining the traditional MD pattern is crucial for public health; in this way, more research is needed to be conducted in order to precisely measure these associations.

## Figures and Tables

**Table 1:** The main baseline of sociodemographic and lifestyle characteristics of the study, *n* = 719.

	All *n* = 719	Women *n* = 346	Men *n* = 373	*P* value
Age, years^*∗*^	38.99 ± 15.31	40.90 ± 14.48	37.22 ± 15.86	0.001
Weight (kg)^*∗*^	71.43 ± 14.09	72.57 ± 15.34	70.38 ± 12.75	0.037
Height (m)^*∗*^	1.66 ± 0.10	1.60 ± 0.08	1.71 ± 0.08	<0.0001
BMI (kg/m^2^)^*∗*^	26.05 ± 6.27	28.26 ± 6.75	24.00 ± 6.75	<0.0001
Age group^*∗∗*^				0.005
≤29	34.1 (245)	28.6 (99)	39.1 (146)	
30–45	33.0 (237)	33.5 (116)	32.4 (121)	
≥46	33.0 (237)	37.9 (131)	28.4 (106)	
Marital status^*∗∗*^				0.001
Not married	48.5 (349)	41.9 (145)	54.7 (204)	
Married	51.5 (370)	58.1 (201)	45.3 (169)	
Occupation^*∗∗*^				<0.0001
With job	65.0 (467)	48.8 (169)	79.9 (298)	
Without job	35.0 (252)	51.2 (177)	20.1 (75)	
Education^*∗∗*^				<0.0001
Illiterate	18.2 (131)	26.6 (92)	10.5 (39)	
<6 years	20.4 (147)	21.4 (74)	19.6 (73)	
≥6 years	61.3 (441)	52.0 (180)	70.0 (261)	
Housing^*∗∗*^				0.175
Traditional housing	2.6 (19)	2.9 (10)	2.4 (9)	
Luxurious	3.6 (26)	4.6 (16)	2.7 (10)	
Flat	53.4 (384)	50.3 (174)	56.3 (210)	
Modern	24.5 (176)	23.7 (82)	25.2 (94)	
Poor housing	15.9 (114)	18.5 (64)	13.4 (50)	
Smoking^*∗∗*^				<0.0001
Current smoker	18.3 (131)	3.5 (12)	32.2 (119)	
Ex-smoker	9.1 (65)	3.5 (12)	14.3 (53)	
Never smoker	72.6 (520)	93.0 (322)	53.5 (198)	
Alcohol consumption^*∗∗*^				<0.0001
Consumer	6.8 (49)	1.7 (6)	11.5 (43)	
Nonconsumer	93.2 (670)	98.3 (340)	88.5 (330)	
BMI class^*∗∗*^				<0.0001
Underweight	5.8 (42)	4.6 (16)	7.0 (26)	
Normal	43.5 (313)	30.6 (106)	55.5 (207)	
Overweight	29.6 (213)	29.8 (103)	29.5 (110)	
Obesity	21.0 (151)	35.0 (121)	8.0 (30)	

^*∗*^ *T*-student test (mean ± standard deviation), ^*∗∗*^chi^2^ test (percent (number)), BMI: body mass index. Statistically significant differences are defined as *P* < 0.05.

**Table 2:** The mean values of usual consumption of major foods or food groups among men and women, *n* = 719.

Dietary variable	Total *n* = 719	Women *n* = 346	Men *n* = 373	*P* value
Daitry products^*∗*^	2.16 (1.25)	2.14 (1.29)	2.14 (1.21)	0.682
Vegetables^*∗*^	4.97 (2.15)	5.16 (2.28)	4.80 (1.99)	0.024
Fruits^*∗*^	2.69 (1.54)	2.79 (1.61)	2.60 (1.47)	0.106
Cereals^*∗*^	4.25 (1.89)	4.25 (2.18)	4.24 (1.57)	0.721
Potatoes^*∗*^	0.78 (0.41)	0.76 (0.45)	0.80 (0.37)	0.153
Beverages^*∗*^	3.26 (2.67)	3.03 (2.55)	3.47 (2.78)	0.026
Sweets^*∗*^	1.40 (1.60)	1.31 (1.60)	1.49 (1.60)	0.151
Legumes^*∗∗*^	4.63 (3.87)	4.32 (3.47)	4.93 (4.20)	0.035
Fish^*∗∗*^	3.33 (3.30)	2.91 (2.91)	3.73 (3.71)	0.001
White meat^*∗∗*^	4.57 (3.94)	4.66 (3.94)	4.48 (3.94)	0.530
Red meat^*∗∗*^	2.52 (2.39)	2.11 (2.10)	2.90 (2.57)	≤0.001
Eggs^*∗∗*^	4.15 (3.18)	3.87 (2.84)	4.41 (3.45)	0.022
Processed meat^*∗∗*^	1.05 (0.12)	0.85 (0.11)	1.24 (0.0.12)	0.023
Soda^*∗∗*^	1.90 (0.25)	1.52 (0.26)	2.25 (0.16)	0.016

^*∗*^Frequency of consumption per day. ^*∗∗*^Frequency of consumption per week. Dairy products (milk, yogurt, and cheese), cereals (bread, cereals, rice, pasta, and couscous), beverages (coffee, tea, and herbal infusions), sweets (Sugar, jelly, candies, pastries, and sweetened fruit juices), white meat (poultry and turkeys), and red meat (veal, lamb, camel, and goat). Variables are presented as mean (standard deviation). Statistically significant differences are defined as *P* < 0.05.

**Table 3:** The mean values of simplified Mediterranean dietary score among the population studied.

	Mean (standard deviation)	*P* value
Total	4.79 (1.39)	
Sex		0.319
Women	4.74 (1.48)	
Men	4.84 (1.31)	
Age group		0.035
≤29	4.61 (1.28)	
30–45	4.89 (1.45)	
≥46	4.90 (1.44)	
Marital status		0.002
Not married	4.63 (1.37)	
Married	4.94 (1.40)	
Occupation		0.557
With job	4.82 (1.39)	
Without job	4.75 (1.41)	
Education		0.149
Illiterate	4.84 (1.37)	
<6 years	4.59 (1.47)	
≥6 years	4.85 (1.37)	
Housing		0.110
Traditional housing	4.63 (1.42)	
Luxurious	5.50 (1.10)	
Flat	4.81 (1.36)	
Modern	4.73 (1.46)	
Poor housing	4.71 (1.43)	
Smoking		0.539
Current smoker	4.68 (1.38)	
Ex-smoker	4.75 (1.35)	
Never smoker	4.83 (1.41)	
Alcohol consumption		0.144
Consumer	5.08 (1.27)	
Nonconsumer	4.77 (1.40)	
BMI class		0.071
Underweight	4.66 (1.07)	
Normal	4.73 (1.38)	
Overweight	4.72 (1.37)	
Obesity	4.06 (1.53)	

BMI: body mass index. Variables are presented as mean (standard deviation).

**Table 4:** Distribution of the daily dietary intake of food groups in relation to simplified Mediterranean-dietary score, *n* = 719.

Dietary variable	Women *n* = 346				Men *n* = 373			
	All	Low-diet score 1–4% (*n*) 42.8 (148)	High-diet score 5–8% (*n*) 57.2 (198)	*P* value	All	Low-diet score 1–4% (*n*) 38.3 (143)	High-diet score 5–8% (*n*) 61.7 (230)	*P* value
Vegetables								
Median	4.73			<0.0001	4.60			<0.0001
≥Median		20.3 (30)	72.2 (143)			25.2 (36)	65.7 (151)	
<Median		79.7 (118)	27.8 (55)			74.8 (107)	34.3 (79)	
Legumes				<0.0001				<0.0001
Median	0.42				0.42			
≥Median		43.9 (65)	78.8 (156)			60.1 (86)	84.8 (195)	
<Median		56.1 (83)	21.2 (42)			39.9 (57)	15.2 (35)	
Fruits				<0.0001				<0.0001
Median	2.71				2.46			
≥Median		20.3 (30)	76.3 (151)			25.5 (35)	66.1 (152)	
<Median		79.7 (118)	23.7 (47)			75.5 (108)	33.9 (78)	
Cereals				<0.0001				<0.0001
Median	4.85				4.85			
≥Median		31.8 (47)	68.2 (135)			29.4 (42)	65.7 (151)	
<Median		61.8 (101)	31.8 (63)			70.6 (101)	34.3 (79)	
Fish				<0.0001				<0.0001
Median	0.28				0.42			
≥Median		45.3 (67)	86.4 (171)			32.2 (46)	65.2 (150)	
<Median		54.7 (81)	13.6 (27)			67.8 (97)	34.8 (80)	
Dairy products				0.475				0.009
Median	2.14				2.14			
≥Median		52.2 (78)	56.6 (112)			57.3 (82)	56.5 (130)	
<Median		47.3 (70)	43.4 (86)			42.6 (61)	43.5 (100)	
Meat				<0.0001				<0.0001
Median	0.85				0.85			
≥Median		63.5 (94)	42.9 (85)			52.4 (75)	31.3 (72)	
<Median		36.8 (54)	57.1 (113)			47.6 (68)	68.7 (158)	
Olive oil				<0.0001				<0.0001
Consumer		85.8 (127)	99.5 (197)			86.7 (124)	97.4 (224)	
Non consumer		14.2 (21)	0.5 (1)			13.3 (19)	2.6 (6)	

**Table 5:** Food groups' consumption according to the categories of adherence to Mediterranean diet.

Adherence to MD	High adherence *n* = 428	Low adherence *n* = 291	*P* value
Food Groups			
Dairy products^*∗*^	2.08 (1.12)	2.22 (1.31)	0.005
Vegetables^*∗*^	5.61 (2.17)	4.04 (1.73)	<0.0001
Fruits^*∗∗*^	3.21 (1.51)	1.94 (1.25)	<0.0001
Cereals^*∗*^	4.56 (1.50)	3.79 (2.27)	<0.0001
Legumes^*∗*^	0.75 (0.54)	0.52 (0.53)	<0.0001
Fish^*∗*^	0.57 (0.53)	0.33 (0.35)	<0.0001
Meat^*∗*^	0.95 (0.65)	1.02 (0.61)	0.001
Olive oil^*∗∗∗*^	98.4%	86.3%	<0.0001

^*∗*^Frequency of consumption per week, ^*∗∗*^number of portions per week. ^*∗∗∗*^Percentage of consumption of olive oil. Variables (^*∗*^and^*∗∗*^) are presented as mean (standard deviation). Statistically significant differences are defined as *P* < 0.05.

**Table 6:** Associations between high adherence to Mediterranean diet and sociodemographic and lifestyle factors, *n* = 719.

	*P* value	Odds ratio	95% CI
Sex			
Women (ref)		1	
Men	0.046	1.46	1.00–2.12
Age group			
≤29	0.085	0.66	0.41–1.05
30–45	0.411	0.84	0.56–1.26
≥46 (ref)		1	
Marital status			
Not married (ref)		1	
Married	0.037	1.46	1.02–2.10
Occupation			
With job	0.260	1.22	0.85–1.75
Without job (ref)		1	
Education			
Illiterate	0.055	1.62	0.98–2.67
>6 years	0.002	1.94	1.27–2.95
≤6 years (ref)		1	
Housing			
Traditional housing	0.974	0.98	0.35–2.73
Luxurious	0.025	3.74	1.18–11.87
Flat	0.965	0.99	0.62–1.56
Modern	0.958	1.01	0.61–1.67
Poor housing (ref)		1	
Smoking			
Current smoker (ref)		1	
Ex-smoker	0.945	0.97	0.51–1.86
Never smoker	0.071	1.57	0.96–2.57
Alcohol consumption			
Consumer	0.025	2.30	1.11–4.79
Nonconsumer (ref)		1	
BMI class			
Underweight	0.633	0.82	0.34–1.80
Normal	0.245	0.75	0.46–1.21
Overweight	0.027	0.59	0.37–1.94
Obesity (ref)		1	

BMI: body mass Index. Statistically significant differences are defined as *P* < 0.05.

## Data Availability

The data used to support the findings of this study are available from the corresponding author upon request.
